# Preliminary study assessing the long-term surgical outcomes of TBX6-associated congenital scoliosis (TACS) patients using the propensity score matching method: exploring the clinical implications of genetic discoveries in congenital scoliosis

**DOI:** 10.1186/s13023-024-03471-9

**Published:** 2025-01-20

**Authors:** Guanfeng Lin, Yang Yang, Zefu Chen, Sen Zhao, Yuchen Niu, You Du, Yiwei Zhao, Shengru Wang, Nan Wu, Jianguo Zhang

**Affiliations:** 1 Department of Orthopaedic Surgery, Peking Union Medical College Hospital (PUMCH), 1St Shuai Fu Yuan, Dongcheng District, Beijing, 100730 People’s Republic of China; 2 Division of Spine Surgery, Department of Orthopedics, Nanfang Hospital, Southern Medical University, Guangzhou, People’s Republic of China; 3 Medical Research Center, Peking Union Medical College and Chinese Academy of Medical Sciences, Peking Union Medical College Hospital, Beijing, 100730 People’s Republic of China

**Keywords:** Congenital scoliosis, TBX6, Prognosis of surgery, Propensity-score matching study, Translational medicine

## Abstract

**Background:**

Compound inheritance of *TBX6* accounts for approximately 10% of sporadic congenital scoliosis (CS) cases. Such cases are called *TBX6*-associated congenital scoliosis (TACS). TACS has been reported to have certain common clinical phenotypes. However, whether the surgical outcomes of TACS patients differ from those of other CS patients remains unclear.

**Methods:**

We retrospectively searched for patients who were diagnosed with scoliosis. TACS was identified in genetic testing for CS. After propensity score matching, patients with TACS were matched with patients with NTACS according to sex, age, main curvature, classification, deformity location, surgical methods, fusion segment and number of fusions. We evaluated and compared the coronal and sagittal radiographic parameters before surgery, immediately after surgery, and at the final follow-up. Surgical information, including surgical method, fusion segment, blood loss and complications, was also compared and analyzed.

**Results:**

Twenty-eight TACS patients were propensity score matched with 28 NTACS patients among 473 CS patients. The preoperative matching parameters mentioned in the Methods section were similar between the TACS group and the NTACS group. In the TACS group, the correction rate of the cranial compensatory curve (64.9 ± 18.6% vs. 51.2 ± 24.0%, P = 0.014) and the correction rate of the caudal compensatory curve (77.4 ± 12.5% vs. 65.4 ± 22.7%, P = 0.011) were significantly greater than those in the NTACS group, and the loss rate of correction of the cranial compensatory curve in the TACS group (0.6 ± 19.2% vs. 26.7 ± 50.8, P = 0.002) was significantly lower than that in the NTACS group. The total complication rate (7.2% vs. 14.3%) and incidence of adding-on (0 vs. 7.1%) were lower in the TACS group than in the NTACS group. There were no significant differences between the two groups in terms of blood loss, revision rate, other correction parameters, balance parameters or incidence of complications.

**Conclusions:**

TACS patients had better surgical outcomes than NTACS patients, which means that genetic diagnosis of the *TBX6* gene mutation in CS before surgery can help predict better surgical outcomes. The specific genetic mechanism is not yet clear and may be related to the relatively normal development of paravertebral tissues in TACS patients. Further research is needed.

**Level of evidence:**

Leve: III.

## Introduction

Congenital scoliosis (CS) is a developmental deformity of vertebrae caused by abnormal somitogenesis in the early stage of embryonic development [1]. The incidence in newborns is approximately 1 per 1000. Previous studies have reported that the incidence of congenital defects of other systems in patients with CS is approximately 50%; such defects are particularly common in the intraspinal canal, cardiovascular system, urinary system and digestive tract system [2]. CS onset often occurs at a very young age and presents with severe deformity, and CS is associated with rapid progression and many comorbidities. If a patient is not diagnosed and treated in time, the deformity will gradually worsen with increasing age and physical growth, which will affect cardiopulmonary development and even neurological function, seriously affecting quality of life and survival. CS may be identified by clinical symptoms such as uneven shoulder height and low back pain. Vertebral development can be divided into three types: failure of formation, failure of segmentation, and mixed type. Conservative treatments such as braces have limited effectiveness, and surgery is the main treatment in the clinic. The main purpose of therapy is to correct the deformity, maintain trunk balance and avoid spine, pulmonary and neurologic defects as much as possible [3]. The occurrence of CS is related to genetic and environmental factors such as hypoxia and vitamin deficiency. In terms of genetics, the most widely accepted model of somitogenesis development is the clock–wavefront model; somitogenesis is mainly regulated by three signaling pathways, namely, the WNT, Notch and FGF pathways, and their corresponding genes and effectors [4]. These three signaling pathways are segmentally activated and expressed during the process of mesoderm formation and subsequently regulate the process of somatoderm formation. Mutations in Notch, FGF and WNT pathway components lead to defects in paraxial mesoderm or somite formation and contribute to CS [5].

The *TBX6* gene encodes the transcription factor T-box 6, a member of the T-box family that plays an important role in the regulation of the development process [6]. TBX6 is localized to 16p11.2, is 6,095 bp in length, contains 8 exons and encodes a 1.9-kb transcript. At 8.5 days post coitus (DPC), Tbx6 transcripts can be detected in the presomitic mesoderm (PSM) of the tail portion (unsegmented) surrounding the caudal end of the neural plate. In the WNT pathway, Wnt3a induces the expression of downstream β-cat, which activates the expression of the downstream axis inhibition protein 2 (AXIN2) and the soluble inhibitor Dickkopf-related protein 1 (DKK1). *Tbx6* is also induced by Wnt3a, which further activates the expression of the *Notch ligand Delta-like gene 1 (DLL1)* and connects with the downstream transcriptional effector Notch intracellular domain (NICD) through *Hes7*, thus establishing a communication loop between the Notch and WNT pathways. Mutations in *TBX6* lead to defects in somite formation and cause CS [7].

Previous studies identified a new genetic model involving a combination of rare null mutations in *TBX6* and common hypomorphic alleles that causes approximately 10% of sporadic CS cases [8]. This type of CS is called *TBX6*-associated congenital scoliosis (TACS). Most TACS patients have certain common clinical features, including vertebral formation failure (hemivertebrae or butterfly vertebrae) and deformities in the lower spine that involve fewer segments. Moreover, the incidence of rib and intramedullary malformations is low in TACS patients [9]. Despite this specific clinical phenotype, there is no relevant literature on the long-term surgical outcomes of TACS patients. Therefore, this study evaluates the surgical outcomes of TACS patients through the propensity score matching method and explores the clinical translational of genetic research on CS.

## Materials and methods

### Subject recruitment

**Fig. 1:**
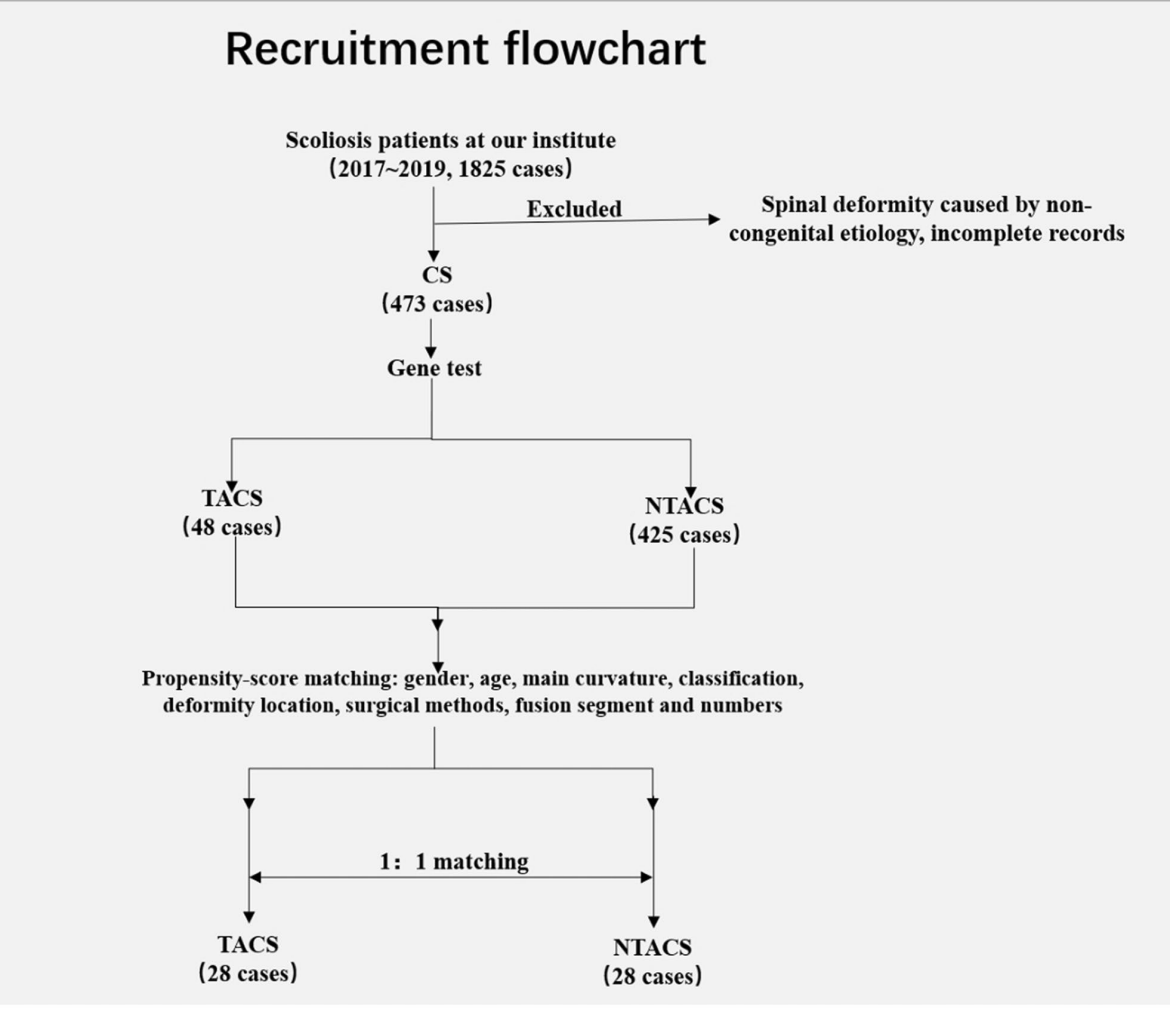
The flowchart presents the recruitment and matching process for patients in both the TACS and NTACS groups

The subjects included in this study were patients with sporadic CS who were admitted to our institute from January 2017 to December 2019. With the approval of the hospital ethics committee, informed consent was obtained from each participant or their guardian, clinical data were collected, and samples were collected. The inclusion criteria were as follows: (1) medical history and examination results consistent with a confirmed clinical diagnosis of congenital scoliosis with the relevant ICD-10 code (Q67.501, Q67.502, Q76.3); (2) complete clinical medical records and examination results; (3) informed consent from the patient and/or guardian; (4) previous one-stage posterior-only spine fusion surgery; and (5) a postoperative follow-up time of more than 24 months. The exclusion criteria were as follows: (1) spinal deformities caused by other reasons, such as congenital or connective tissue diseases or genetic syndromes; (2) conservative treatment and/or other surgeries (such as the anterior approach or nonfusion surgery); and (3) clinical manifestations meeting the diagnostic criteria for syndromic scoliosis, such as Jarcho-Levin syndrome, Klippel-Feil syndrome and Freeman-Shelton syndrome. The recruitment flowchart is shown in Fig. 1.

### Genetic testing

The QIAamp DNA Blood Maxi Kit (QIAGEN) was used to extract DNA from peripheral blood (5–7 ml) from the included CS patients. The exome was enriched using Agilent's All Exon V6 + UTR r2. An Illumina double-terminal library was constructed from the DNA samples of patients and family members. The generated raw data were analyzed using the self-developed Peking Union Medical College Hospital Pipeline (PUMP) for mutation identification and annotation. The aligned reads were realigned and recalibrated by the GATK Indel Realigner and the GATK Base Recalibrator, respectively (broadinstitute.org/). The GATK UnifiedGenotyper (broadinstitute.org/) was used to identify single‐nucleotide variants (SNVs) and small insertions or deletions (indels) [10–12]. Variants were filtered and selected based on a minor allele frequency with a cutoff value of < 0.05 in three databases (1000 Genomes Project, NHLBI GO Exome Sequencing Project and Exome Aggregation Consortium) [10–12]. A variety of bioinformatics prediction tools (Gerp + + , Combined Annotation Dependent Depletion, SIFT, PolyPhen-2 and VariantTaster) were used to predict the conservation and pathogenicity of candidate mutations. Variants were classified according to the variant interpretation guidelines of the American College of Medical Genetics and Genomics (ACMG) [13]. Sanger sequencing was performed to validate the variants identified by whole-exome sequencing. TBX6 sequence NM_004608.4 was used as the reference sequence.

### Clinical evaluation and radiographic measurement

All enrolled patients underwent X-ray before surgery, immediately after surgery (within 2 weeks), and at the final follow-up. All patients underwent spinal CT, spinal MRI, echocardiography, abdominal ultrasound, urinary ultrasound and gynecological ultrasound examinations. Clinical information, including basic demographics (age, sex, ethnicity, etc.), deformity-related conditions (Winter classification, vertebral body involvement, intraspinal malformations, etc.), surgical information (surgery methods, osteotomy methods, osteotomy segments, fusion segments, bleeding, complications such as adding-on, junctional kyphosis and crankshaft phenomenon, etc.) and hospitalization information, was recorded.

The following radiographic parameters were measured: main curve (the maximum angle between the superior endplate of the most tilted vertebra cranially and the inferior endplate of the most tilted vertebra caudally), compensatory cranial curve (the cranial Cobb angle of the main curves), compensatory caudal curve (the caudal Cobb angle of the main curves), segmental kyphosis (the angle of one segment above and below the hemivertebra), coronal balance (the perpendicular distance between the C7 plumb line and the central sacral vertical line), sagittal balance (the distance between the plumb line dropped from the center of C7 and the posterior superior corner of S1), thoracic kyphosis (the angle between the superior endplate of T5 and the inferior endplate of T12), thoracolumbar kyphosis (the angle between the superior endplate of T10 and the inferior endplate of L2) and lumbar lordosis (the angle between the superior endplate of L1 and the inferior endplate of L5). All data were recorded by two senior orthopedic surgeons, and the average value was used as the final result.

### Propensity score matching

Patients identified as having *TBX6* mutations were assigned to the TACS group, and the other patients were classified into the non-TACS (NTACS) group. With the help of the Matchit_n function of R software, 1:1 propensity score matching between the TACS and NTACS groups was conducted according to the following parameters: ① age (a difference of less than 1 year), ② sex (same sex), ③ Cobb angle of the curve deformity (a difference of less than 5°), ④ CS classification (same Winter classification), ⑤ location of the major deformity (within 2 segments), ⑥ surgical methods (vertebrotomy, follow-up duration, blood loss, number of fused segments), ⑦ fusion level (difference between the upper and lower fixed vertebrae is within 2 segments), ⑧ number of fused segments (within 2 segments), ⑨ presence of intraspinal deformity or not. The flowchart of recruitment and matching is shown in Fig. 1.

### Statistical analysis

Descriptive statistics are presented as the mean ± standard deviation for continuous variables and categorical variables. Frequency is used to describe categorical variables. The chi-square test or Fisher’s exact probability test was used to evaluate the relationships between categorical variables. Paired t tests or nonparametric tests were used to compare continuous variables between groups. A P value < 0.05 was considered to indicate statistical significance. IBM SPSS Statistics 23 was used for the statistical analysis.

## Results

### Genetic testing results

This study enrolled 473 patients with sporadic CS, in which 48 (10.1%) were assigned to the TACS group (37 with 16p11.2 duplication deletion and 11 with nonsense mutations with T-C-A hypomorphic haploids in the other allele), and the remaining 425 patients were assigned to the NTACS group.

Twenty-eight pairs of patients were matched by propensity score matching. Among the 28 matched TACS patients, 16p11.2 deletion copy number variation (CNV) was identified by exon analysis and verified by a CGH exam in 21 patients, while the remaining 7 patients had TBX6 segmental alleles (frameshift mutation, stop gain or splice region variation). The TBX6 risk haplotype of T-C-A (defined by the nonreference alleles of three common single-nucleotide polymorphisms [SNPs], rs2289292, rs3809624, and rs3809627) was detected in all 28 patients. Genetic characteristics of patients with TBX6-Associated Congenital Scoliosis is shown in Table 1.

**Table 1:** Genetic Characteristics of Patients with TBX6-Associated Congenital Scoliosis

Patient No	TBX6 Allele	
	Null Mutation at First Allele	Risk Haplotype at Second Allele
1	c.266dupC(p.Val91GlyfsTer80)	T-C-A
2	c.1169dupC(p.His391AlafsTer96)	T-C-A
3	c.1061delC(p.Ala354GlyfsTer144)	T-C-A
4	c.1179_1180delAG(p.Gly395LeufsTer91)	T-C-A
5	c.621 + 1G > A	T-C-A
6	c.1121_1122del(p.Pro374ArgfsTer112)	T-C-A
7	c.903 T > G(p.Tyr301Ter)	T-C-A
8	16p11.2 del	T-C-A
9	16p11.2 del	T-C-A
10	16p11.2 del	T-C-A
11	16p11.2 del	T-C-A
12	16p11.2 del	T-C-A
13	16p11.2 del	T-C-A
14	16p11.2 del	T-C-A
15	16p11.2 del	T-C-A
16	16p11.2 del	T-C-A
17	16p11.2 del	T-C-A
18	16p11.2 del	T-C-A
19	16p11.2 del	T-C-A
20	16p11.2 del	T-C-A
21	16p11.2 del	T-C-A
22	16p11.2 del	T-C-A
23	16p11.2 del	T-C-A
24	16p11.2 del	T-C-A
25	16p11.2 del	T-C-A
26	16p11.2 del	T-C-A
27	16p11.2 del	T-C-A
28	16p11.2 del	T-C-A

### Comparison of matching parameters between the two groups before surgery

The basic conditions and parameters of the two groups before surgery are shown in Table 2. There were no significant differences in age, sex, Winter classification, number of fused segments, preoperative main curve angle, compensatory cranial curve, compensatory caudal curve, thoracic kyphosis, thoracolumbar kyphosis, lumbar lordosis, coronal balance, or sagittal balance between the two groups (all p > 0.05). The surgical evaluation comparison results are shown in Table 3. There were no significant differences in the number of fusion segments, blood loss, revision rate, or follow-up duration between the two groups (all p > 0.05).

**Table 2:** Comparison of preoperatively and surgically matching parameters between the two groups

	TACS	NTACS	*p* value
Preoperatively matching parameters			
Age	4.139 ± 2.887	4.289 ± 2.864	0.846
Male (%)	19 (67.9%)	19 (67.9%)	1
Failure of formation (%)	25 (89.3%)	25 (89.3%)	1
Main Curve	43.8 ± 10.6	41.5 ± 9.3	0.386
Compensatory cranial curve	19.5 ± 9.0	19.4 ± 5.7	0.935
Compensatory caudal curve	22.8 ± 6.4	22.2 ± 7.3	0.735
Segmental kyphosis	29.7 ± 18.3	25.5 ± 18.7	0.393
Thoracic Kyphosis	29.2 ± 15.5	30.1 ± 10.4	0.798
Thoracolumbar Kyphosis	23.1 ± 18.5	15.0 ± 17.0	0.093
Lumbar lordosis	46.8 ± 12.9	40.6 ± 14.1	0.09
Coronal balance	13.5 ± 7.4	13.3 ± 7.1	0.892
Sagittal balance	− 1.1 ± 29.2	− 0.854 ± 25.5	0.973
Surgically matching parameters			
Follow-up duration	40.4 ± 19.7	43.5 ± 15.0	0.568
Blood loss	232.5 ± 121.3	216.8 ± 127.1	0.638
Number of fused segments	4.4 ± 1.5	4.3 ± 1.5	0.791

**Table 3:** Comparison of surgical and correction parameters between the two groups

	TACS	NTACS	*p* value
Main curve			
Preoperative (°)	43.8 ± 10.6	41.5 ± 9.3	0.386
Postoperative (°)	9.1 ± 6.1	9.1 ± 5.3	0.978
Final follow-up (°)	11.1 ± 6.6	13.5 ± 9.7	0.191
Correction rate (%)	76.0 ± 9.6	68.2 ± 21.1	0.053
Compensatory cranial curve			
Preoperative (°)	19.5 ± 9.0	19.4 ± 5.7	0.935
Postoperative (°)	7.1 ± 6.8	6.9 ± 4.8	0.894
Final follow-up (°)	7.5 ± 6.5	9.7 ± 6.6	0.161
Correction rate (%)	64.9 ± 18.6	51.2 ± 24.0	0.014*
Compensatory caudal curve			
Preoperative (°)	22.8 ± 6.4	22.2 ± 7.3	0.735
Postoperative (°)	5.3 ± 4.1	5.7 ± 4.3	0.709
Final follow-up (°)	5.4 ± 4.0	7.5 ± 5.9	0.025*
Correction rate (%)	77.4 ± 12.5	65.4 ± 22.7	0.011*
Coronal balance			
Preoperative (mm)	13.5 ± 7.4	13.3 ± 7.1	0.892
Postoperative (mm)	9.6 ± 5.4	10.8 ± 7.4	0.486
Final follow-up (°)	7.4 ± 4.0	11.0 ± 9.8	0.080

^*^P < 0.05

### Comparison of coronal parameters after surgery and at the last follow-up

The coronal parameters after surgery and at the final follow-up are shown in Table 3. At the final follow-up, the correction rates of compensatory cranial curvature (64.9 ± 18.6% vs. 51.2 ± 24.0%, P = 0.014) and compensatory caudal curvature (77.4 ± 12.5% vs. 65.4 ± 22.7%, P = 0.011) were significantly greater in the TACS group than in the NTACS group. There were no significant differences in the other coronal parameters between these two groups (all p > 0.05).

### Comparison of the sagittal parameters after surgery and at the last follow-up

The balance parameters of the two groups are shown in Table 4. There were no significant differences in postoperative and final follow-up parameters between the two groups in terms of thoracic kyphosis, thoracolumbar kyphosis, lumbar lordosis, coronal balance, or sagittal balance (all p > 0.05).

**Table 4:** Comparison of sagittal parameters between the two groups

	TACS	NTACS	p value
Segmental kyphosis (°)			
Preoperative	29.7 ± 18.3	25.5 ± 18.7	0.393
Postoperative	6.4 ± 9.4	4.6 ± 9.6	0.471
Final follow-up	7.2 ± 9.6	7.8 ± 15.9	0.780
Sagittal balance (mm)			
Preoperative	−1.1 ± 29.2	−0.854 ± 25.5	0.973
Postoperative	7.6 ± 21.3	3.2 ± 20.2	0.436
Final follow-up	−2.6 ± 21.5	−2.8 ± 23.2	0.984
Thoracic kyphosis (°)			
Preoperative	29.2 ± 15.5	30.1 ± 10.4	0.798
Postoperative	25.8 ± 8.3	28.2 ± 7.7	0.275
Final follow-up	28.6 ± 6.9	32.4 ± 8.5	0.064
Thoracolumbar kyphosis (°)			
Preoperative	23.1 ± 18.5	15.0 ± 17.0	0.093
Postoperative	5.1 ± 6.8	5.0 ± 6.4	0.952
Final follow-up	6.0 ± 5.8	7.2 ± 6.8	0.481
Lumbar lordosis (°)			
Preoperative	46.8 ± 12.9	40.6 ± 14.1	0.09
Postoperative	40.7 ± 10.2	42.8 ± 11.4	0.466
Final follow-up	45.6 ± 7.9	45.5 ± 9.5	0.966

### Comparison of complications

A comparison of complications between the two groups is shown in Table 5. Although there were no statistically significant differences, the total incidence of complications (7.2% vs. 14.3%), the total incidence of correction loss (3.6% vs. 10.7%) and the incidence of adding-on (0 vs. 7.1%) were significantly lower in the TACS group than in the NTACS group.

**Table 5:** Comparison of complications between the two groups

	TACS	NTACS	*p* value
Total complications (n (%))	2(7.2%)	4(14.3%)	0.669
Implant-related (n (%))	0	0	1
Neurological damage (n (%))	0	0	1
Correction loss related (n (%))	1 (3.6%)	3 (10.7%)	0.611
Adding-on (n (%))	0	2 (7.1%)	0.491
Crankshaft (n (%))	0	0	1
PJK (n (%))	1 (3.6%)	1 (3.6%)	1
DJK (n (%))	0	0	1
Incision-related (n (%))	1 (3.6%)	1 (3.6%)	1
Hemopneumothorax (n (%))	0	0	1
Pseudoarthrosis (n (%))	0	0	1

## Discussion

CS manifests early in life, is characterized by rigidity and rapid progression, and often necessitates surgical intervention [1]. CS is caused by both genetic and environmental factors [5]. The current genetic pathogenic framework explains only approximately 20% of CS cases, and half of these cases are explained by the *TBX6* gene, making *TBX6* the most significant genetic etiology [8]. A previous study revealed that common clinical features of TACS include vertebral formation failure (e.g., hemivertebrae or butterfly vertebrae), deformities in the lower spine (T8-S1), the involvement of fewer segments, ribs with normal shapes or only slight deformities, and a lower incidence of intraspinal malformations [9]. Based on this specific clinical phenotype, we speculated that certain surgical outcomes may prove more favorable for TACS patients than for other CS patients. The objective of this study was to investigate the long-term surgical outcomes of TACS patients by using a strict matching method. We found that TACS patients had superior outcomes compared with their NTACS counterparts. These findings highlight the potential for clinical translational application of genetic research achievements in the field of CS.

Wu et al. [8] first showed that the new compound genetic mechanism (rare CNV + common SNP) of the *TBX6* gene accounted for 10.6% (17/161) of CS cases. Liu et al. [9] further verified this novel pathogenic model in three different races. In this study, 473 patients with CS who underwent genetic testing were included. Among these 473 CS patients, 48 patients with 16p11.2 duplication deletion and *TBX6* nonsense mutations were identified (10.1%), consistent with the proportion reported in previous studies. Overall, 28 TACS patients were successfully matched. As Table 1 shown, the 16p11.2 deletion CNV was identified and verified in 21 patients, whereas the other 7 patients presented with *TBX6* segmental alleles (frameshift mutation, stop-gain or splice region variations). The *TBX6* risk haplotype of T-C-A (SNPs rs2289292, rs3809624, and rs3809627) was observed in all 28 patients, consistent with previous findings on the compound inheritance of TACS [8, 9, 14].

The rigorous 1:1 propensity score case matching procedure accounted for factors such as age, sex, main curve angle, CS clinical classification, segment of the deformity and the presence of intraspinal malformation in line with the specific clinical phenotype of TACS, as summarized in previous studies [9]. Furthermore, to reduce the impact of differences in surgical aspects, we also compared surgical parameters, including surgical method, fused segments, and number of fused segments, between the two groups. As shown in Table 2, there were no significant differences in preoperative matching parameters between the TACS and NTACS groups; in other words, these stringent matching parameters helped to minimize variation in preoperative and surgical conditions and improve the comparability of the two groups. Importantly, this process greatly mitigated the effects of confounding variables and enhanced the validity and robustness of our findings.

**Fig. 2:**
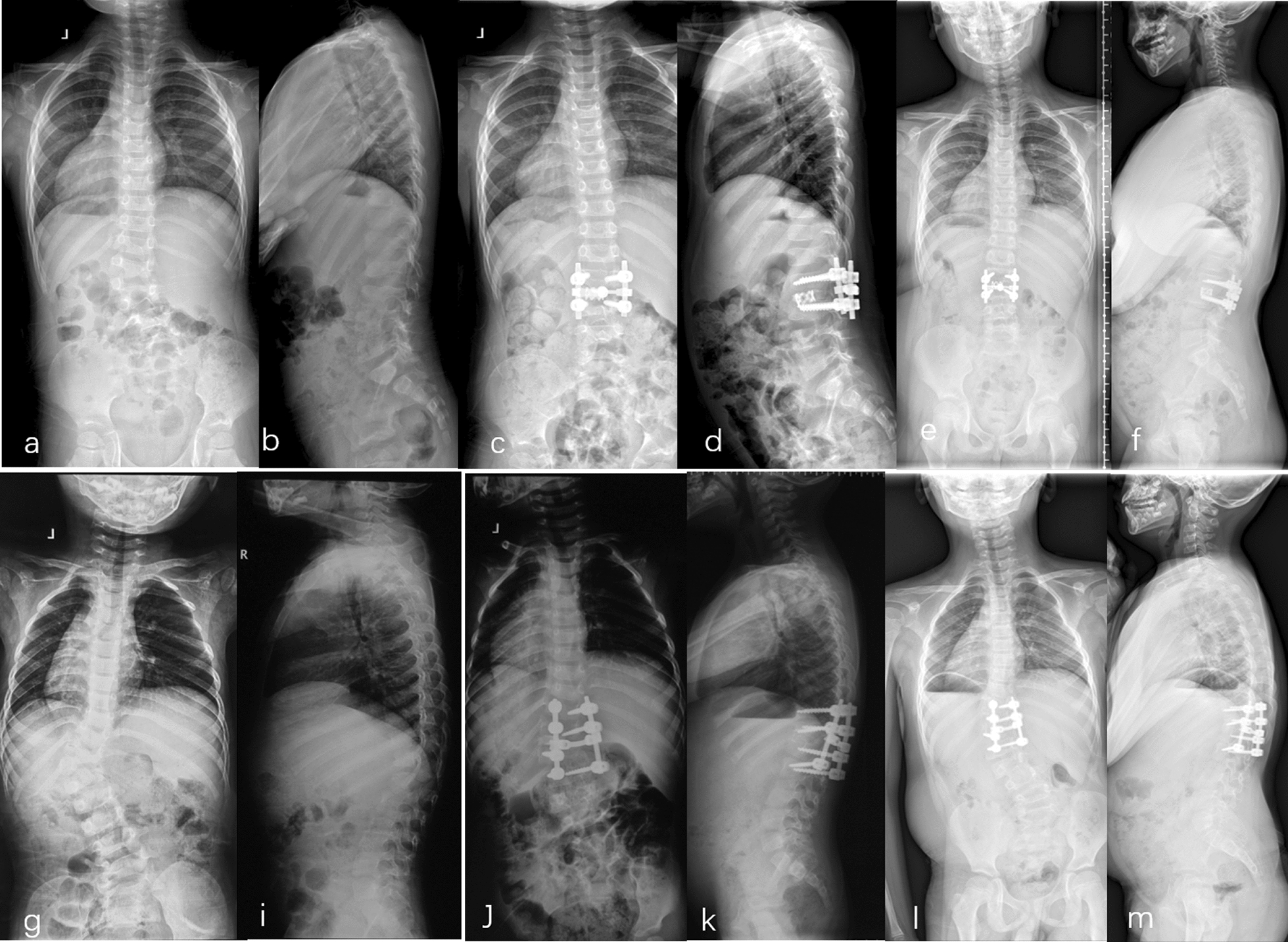
The above part (**a–f**) of this figure refers to a 3-year-old male TACS patient. Genetic testing showed that 16p11.2 was deleted in combination with the TCA hypomorphic allele. (**a**–**b**) show a main curve of L1 butterfly vertebrae, L2 left segmented hemivertebrae, normal ribs, and no obvious intraspinal deformity. He underwent posterior L2 hemivertebral resection, short-segment fixation, and bone graft fusion (L1-3). The postoperative (**c**, **d**) and 48-month final follow-up (**e**, **f**) images showed that the correction and trunk balance were satisfactory. The lower part (**g**–**m**) of this figure refers to the matching NTACS patient. This was a 3-year-old male with a main curve of completely segmented T12/L1 hemivertebrae, normal ribs and no intraspinal deformity (**g**, **i**). He received posterior T12/L1 hemivertebra resection, short-segment fixation, and bone graft fusion (T11-L2). The immediate postoperative correction was good (**j**, **k**). However, at the 50-month final follow-up, the LIV moved to the distal end of the fused segment, and the adding-on phenomenon occurred (**l**, **m**)

Compared with other types of spinal deformities, CS tends to have early onset and rapid progression. In severe cases, it can even cause progressive pulmonary dysfunction and neurological damage. Surgery is still the most common treatment method for CS [2]. In this series, all patients achieved satisfying sagittal balance and no difference between the two groups after surgery and at the last follow-up. This is similar with previous studies [15, 16] and reveals that posterior hemivertebra resection and segment fusion surgery is reliable and effective for CS. This study compared surgical outcomes after surgery and at the last follow-up. The correction rates of the main curve (76.0 ± 9.6% vs. 68.2 ± 21.1%), compensatory cranial curve (64.9 ± 18.6% vs. 51.2 ± 24.0%) and compensatory caudal curve (77.4 ± 12.5% vs. 65.4 ± 22.7%) were significantly higher in the TACS group than in the NTACS group. Moreover, the incidences of total complications (7.2% vs. 14.3%) and the adding-on phenomenon (0 vs. 7.1%) were lower in the TACS group than in the NTACS group. These results revealed that surgical outcomes were better in the TACS group than in the NTACS group. Figure 2 shows such a pair of typical matched cases. Both patients were 3-year-old male patients with a matching main curve, segmental kyphosis, normal ribs, no obvious intraspinal deformity, and hemivertebra in the thoracolumbar region who underwent hemivertebra resection and short-segment fixation. The top portion of the figure shows the TACS patient, and the postoperative(c, d) and 48-month follow-up (e, f) images indicate that the correction and balance were satisfactory. The lower portion of the figure shows an NTACS patient, and the postoperative (j, k) correction performed well; however, at the 50-month follow-up (l, m), he suffered from the distal adding-on phenomenon, thus showing worse surgical outcomes than the matching TACS patient.

We suggest that genetic pathologies underlie the poorer surgical outcomes in the NTACS group compared with the TACS group. We explored the biological processes associated with CS by searching the Gene Ontology database and discovered that the modulation of paravertebral tissues represented prominent pathways among the CS pathogenic genes. On the one hand, the *TBX6* gene plays a major role in paraxial mesoderm differentiation and somite segmentation in early embryonic development [17, 18]. *TBX6* can activate the transcription of Mesp2 and Ripply2 [19, 20], which promotes the development of somites and vertebral bodies [21]. The *TBX6* gene is relatively ‘pure’ and is mainly involved in the formation of vertebral bodies rather than other paravertebral tissues, consistent with the relatively mild clinical phenotype of TACS. On the other hand, the current genetic pathogenic framework explains only approximately 20% of CS cases; NTACS patients have a complex and unclear pathogenesis that may affect both spine and paravertebral tissue development of cartilage, muscles and ligaments and other soft tissues, which will negatively affect surgical outcomes. For example, there are many subtypes of the human *MYH* gene, which encodes the basic unit of the myosin-myosin heavy chain (MyHC). The *MYH* gene plays an important role in ensuring normal muscle cell function, and a previous study reported associations between the *MYH* gene and pathological changes in the development and function of paraspinal muscles and CS [22]. Therefore, the TACS patients had better surgical outcomes than did the matched NTACS patients.

Diseases caused by gene mutations often have corresponding specific clinical manifestations, and a clear genetic diagnosis can guide clinical treatment. Clarifying the relationship between genotype and clinical outcomes is important for the treatment of CS. This study helps clarify and strengthen this relationship because it is the first to use a strict matching method to evaluate the long-term prognosis of a specific population of TACS patients in whom the *TBX6* gene mutation is the clear cause. The current study revealed that genetic diagnosis of the *TBX6* gene mutation in CS before surgery can help predict better surgical outcomes. This study is also the first to investigate the clinical translation of genetic research findings to CS surgical interventions. However, broader studies involving larger cohorts are required to fully elucidate the underlying mechanisms.

## Conclusion

TACS patients had better surgical outcomes than NTACS patients, which means that genetic diagnosis of the *TBX6* gene mutation in CS before surgery can help predict better surgical outcomes. The specific genetic mechanism is not yet clear and may be related to the relatively normal development of paravertebral tissues in TACS patients. Further studies are needed. This study generated novel insights that hold preliminary implications for the clinical translation and practical application of CS pathogenic gene research findings.

## Data Availability

The data that support the findings of this study are not openly available due to reasons of sensitivity and privacy and are available from the corresponding author upon reasonable request. Data are located in controlled access data storage at Peking Union Medical College Hospital.

## Abbreviations

CS: Congenital Scoliosis
TACS: TBX6-associated Congenital Scoliosis
NTACS: Non TBX6-associated Congenital Scoliosis
DPC: Days post coitus
PSM: Presomitic mesoderm
AXIN2: Axis inhibition protein 2
DKK1: Dickkopf-related protein 1
DLL1: Notch ligand Delta-like gene 1
NICD: Notch intracellular domain
SNVs: Single-nucleotide variants
ACMG: American College of Medical Genetics and Genomics
CNV: Copy number variation
SNPs: Single-nucleotide polymorphisms
MyHC: Myosin-myosin heavy chain
